# The incidence of psychotic disorders among migrants and minority ethnic groups in Europe: findings from the multinational EU-GEI study

**DOI:** 10.1017/S0033291720003219

**Published:** 2022-05

**Authors:** Fabian Termorshuizen, Els van der Ven, Ilaria Tarricone, Hannah E. Jongsma, Charlotte Gayer-Anderson, Antonio Lasalvia, Sarah Tosato, Diego Quattrone, Caterina La Cascia, Andrei Szöke, Domenico Berardi, Pierre-Michel Llorca, Lieuwe de Haan, Eva Velthorst, Miguel Bernardo, Julio Sanjuán, Manuel Arrojo, Robin M. Murray, Bart P. Rutten, Peter B. Jones, Jim van Os, James B. Kirkbride, Craig Morgan, Jean-Paul Selten

**Affiliations:** 1 Rivierduinen Institute for Mental Health Care, Sandifortdreef 19, 2333 ZZ Leiden, The Netherlands; 2 Mailman School of Public Health, Columbia University, New York City, USA; 3 Department of Psychiatry and Neuropsychology, School for Mental Health and Neuroscience, Maastricht University Medical Centre, P.O. Box 616, 6200 MD Maastricht, The Netherlands; 4 Department of Medical and Surgical Science, Bologna Transcultural Psychosomatic Team (BoTPT), Alma Mater Studiorum Università di Bologna, Viale Pepoli 5, 40126 Bologna, Italy; 5 Department of Biomedical and Neuro-motor Sciences, Psychiatry Unit, Alma Mater Studiorum Università di Bologna, 40126 Bologna, Italy; 6 Department of Psychiatry, University of Cambridge, Herchel Smith Building for Brain & Mind Sciences, Forvie Site, Robinson Way, Cambridge, CB2 0SZ, UK; 7 Psylife Group, Division of Psychiatry, University College London, 6th Floor, Maple House, 149 Tottenham Court Road, London, W1T 7NF, UK; 8 Department of Health Service and Population Research, Institute of Psychiatry, King's College London, De Crespigny Park, Denmark Hill, SE5 8AF, London, UK; 9 Section of Psychiatry, Department of Neurosciences, Biomedicine and Movement Sciences, University of Verona, Verona, Italy; 10 Department of Psychosis Studies, Institute of Psychiatry, King's College London, De Crespigny Park, Denmark Hill SE5 8AF, London, UK; 11 Unit of Psychiatry, “P. Giaccone” General Hospital, Via G. La Loggia n.1, 90129 Palermo, Italy; 12 INSERM, U955, Equipe 15, 51 Avenue de Maréchal de Lattre de Tassigny, 94010 Créteil, France; 13 CMPB CHU Clermont-Ferrand, EA 7280, University Clermont Auvergne, Clermont-Ferrand, France; 14 Department of Psychiatry, Early Psychosis Section, Amsterdam UMC, location AMC, University of Amsterdam, Meibergdreef 5, 1105 AZ Amsterdam, The Netherlands; 15 Department of Psychiatry and Seaver Autism Center for Research and Treatment, Icahn School of Medicine at Mount Sinai, New York, USA; 16 Barcelona Clinic Schizophrenia Unit, Neuroscience Institute, Hospital Clinic of Barcelona, Barcelona, Spain; 17 Department of Medicine, University of Barcelona, IDIBAPS, CIBERSAM, Barcelona, Spain; 18 Department of Psychiatry, School of Medicine, Universidad de Valencia, Centro de Investigación Biomédica en Red de Salud Mental (CIBERSAM), C/Avda. Blasco Ibáñez 15, 46010 Valencia, Spain; 19 Department of Psychiatry, Psychiatry Genetic Group, Instituto de Investigación Sanitaria de Santiago de Compostela, Complejo Hospitalario Universitario de Santiago de Compostela, 15706 Santiago de Compostela, Spain; 20 CAMEO Early Intervention Service, Cambridgeshire & Peterborough NHS Foundation Trust, Cambridge, CB21 5EF, UK; 21 Department Psychiatry, Brain Center Rudolf Magnus, Utrecht University Medical Centre, Utrecht, The Netherlands

**Keywords:** Dopamine, epidemiology, ethnicity, migration, psychosis, race, schizophrenia, stress

## Abstract

**Background:**

In Europe, the incidence of psychotic disorder is high in certain migrant and minority ethnic groups (hence: ‘minorities’). However, it is unknown how the incidence pattern for these groups varies within this continent. Our objective was to compare, across sites in France, Italy, Spain, the UK and the Netherlands, the incidence rates for minorities and the incidence rate ratios (IRRs, minorities *v.* the local reference population).

**Methods:**

The European Network of National Schizophrenia Networks Studying Gene–Environment Interactions (EU-GEI) study was conducted between 2010 and 2015. We analyzed data on incident cases of non-organic psychosis (International Classification of Diseases, 10th edition, codes F20–F33) from 13 sites.

**Results:**

The standardized incidence rates for minorities, combined into one category, varied from 12.2 in Valencia to 82.5 per 100 000 in Paris. These rates were generally high at sites with high rates for the reference population, and low at sites with low rates for the reference population. IRRs for minorities (combined into one category) varied from 0.70 (95% CI 0.32–1.53) in Valencia to 2.47 (95% CI 1.66–3.69) in Paris (test for interaction: *p* = 0.031). At most sites, IRRs were higher for persons from non-Western countries than for those from Western countries, with the highest IRRs for individuals from sub-Saharan Africa (adjusted IRR = 3.23, 95% CI 2.66–3.93).

**Conclusions:**

Incidence rates vary by region of origin, region of destination and their combination. This suggests that they are strongly influenced by the social context.

## Introduction

Studies in Western Europe have found an increased incidence of affective and non-affective psychotic disorder among various migrant and minority ethnic groups (Bourque, van der Ven, & Malla, 2011; Selten, van der Ven, & Termorshuizen, 2020). Irrespective of minority status, there is prominent heterogeneity in psychosis incidence between places (Jongsma, Turner, Kirkbride, & Jones, 2019). Recent findings from the European Network of National Schizophrenia Networks Studying Gene–Environment Interaction (EU-GEI) study indicated an almost 8-fold variation across 17 sites in six countries ranging from 6.0 (95% CI 3.5–8.6) per 100 000 person-years in Santiago de Compostela, Spain, to 46.1 (95% CI 37.3–55.0) per 100 000 person-years in Paris, France (Jongsma et al., 2018). As identical inclusion criteria and similar methods of assessment were used, data from this study provides a unique opportunity to test whether the absolute and relative risks for minorities vary by site.

Incidence studies are based on numerators and denominators. In most European countries the denominator information is organized around the variable country of birth. This permitted a comparison of risk for first-generation migrants to that for the native-born. In the UK, by contrast, where this information is based on self-assigned ethnicity, we compared the risk for members of ethnic minorities (usually migrants of the first, second or third generation) to that for White British individuals. We use the word ‘minorities’ to designate both migrants and, with reference to the UK, members of ethnic minorities. Thus, our term ‘minorities’ does not refer to religious or sexual minorities. The term ‘reference population’ refers to all other citizens.

To test the null hypothesis that incidence rates of psychosis among minorities would be similarly elevated across European regions, we compared the rates for minorities and the incidence rate ratios (minorities *v.* the local reference populations) across sites. The aim was to assess whether there are differences in the incidence rates and incidence rate ratios (IRRs) across recruitment sites. This was investigated for all minorities together and for seven sub-groups, categorized according to their region of origin.

## Methods

### Study design and setting

EU-GEI is a multi-center incidence and case-control study of gene–environment interactions in psychotic disorder and has been described previously (Jongsma et al., 2018). Briefly, we identified individuals 18–64 years of age who resided within the catchment areas and presented to mental health services for a suspected first episode of psychosis. Potential participants were asked to provide informed consent for an assessment that included a semi-structured diagnostic interview (for details, see online Supplementary eMethods).

We had to exclude the sites Puy de Dôme, Verona, Madrid and Brazil (see online Supplementary eMethods). As for the remaining 13 sites, 82 cases (4.2%) were excluded because information on country of birth or ethnicity was missing or because a case could not be linked to the denominator data.

### Population at risk

The population at risk in each site during the recruitment period was estimated from the most accurate local routine demographic data, stratified by age (5-year bands), sex, country or region of origin or ethnicity.

The statistical bureaus in France, Italy and Spain identify migrants using registered country of birth. Thus, in the catchment areas of these countries, we compared the rate of psychosis for foreign-born individuals to that for native-born individuals. Since denominator information on parental country of birth was lacking, the numbers of second-generation migrants were necessarily included in the reference population.

The classification of region of birth of the French bureau is detailed in the online Supplementary eMethods.

For the Netherlands, we used population data on country of birth and parental countries of birth to distinguish between the native Dutch (Dutch-born individuals born to Dutch-born parents), first- and second-generation migrants. A Dutch-born individual is considered a second-generation migrant if at least one of the parents was born abroad. At Dutch sites, we compared the incidence rate of psychosis for first- and second-generation migrants combined with that for the native Dutch.

For the UK we used data from the 2011 census which recognizes a range of categories (see online Supplementary eMethods), based on self-assigned ethnicity and country of birth (the UK or not). We compared the incidence rate for minorities in the UK to that for the White British. Thus, denominator information on minorities born in the country of destination was available in the Netherlands (second generation) and in the UK (second generation or higher), not in France, Italy or Spain (online Supplementary eTable S1).

### Exposure classification

Given the available information and the geographical and cultural differences, we divided minorities by the following regions of origin: 1. Western countries: Europe, USA, Canada, Australia, New Zealand and countries of the former Soviet Union with a predominant Christian religion (Asian states with a predominant Islamic population were put in category 5.); 2. Middle East (includes also Turkey, Israel and Egypt); 3. The Maghreb (North-African countries, except Egypt); 4. sub-Saharan Africa; 5. Asia (including states of the former Soviet Union with a predominant Islamic population); 6. Latin America; 7. The Caribbean islands (including Martinique and Guadeloupe, the Netherlands Antilles, Jamaica, Barbados, Trinidad and Tobago), Surinam, Guyana, French Guyana (three non-Latin countries in South-America), and the other French overseas departments (the population of the Caribbean is predominantly of African origin, while that of the other regions is mixed). We considered individuals from categories 2 through 7 as non-Western migrants.

This classification did not entirely overlap with that used by the UK census, but several comparisons were possible. The details are summarized in the online Supplementary eMethods.

There were too few people from the Maghreb or Latin America in the UK for meaningful analyses. For this reason, we excluded the UK from the comparison of rates for individuals from these regions. Likewise, persons from the Middle-East in Spain were not included as separate denominator category. Since the French data did not allow the delineation of migrants from the Middle East, Asia, or Latin America, the French sites could not be included in these comparisons.

### Outcome and determinants

Our primary outcome was an OPCRIT-confirmed ICD-10 diagnosis of any psychotic disorder (ICD-10 codes F20–F33). OPCRIT is based on a computer algorithm of rated symptoms and is less prone to diagnostic bias than clinical diagnosis (Rucker et al., 2011). When patients refused to be interviewed, we could retrieve the information required for OPCRIT from the clinical file at all sites, except Gouda & Voorhout, The Netherlands. If there was no OPCRIT available, we relied on clinical diagnosis.

The broad category of any psychotic disorder was subdivided into non-affective (NAPD)(F20–F29) and affective psychotic disorder (APD)(F30–F33). Data on age at first contact, sex, self-ascribed ethnicity, and personal and parental country of birth were collected from interview or case notes using the Medical Research Council Sociodemographic Questionnaire (Mallett, 1997).

### Statistical analysis

We estimated the incidence per 100 000 person years by site for minorities and the reference population. As for sites in France, Italy and Spain, we compared the incidence rates for foreign-born individuals to that for native-born individuals. Since denominator information on parental country of birth was lacking, second-generation migrants were necessarily included in the native-born population. With reference to sites in the UK and in the Netherlands: since meta-analytic work reports small differences in risk between individuals of the first and the second generation, we compared incidence rates between minorities and the reference population without distinguishing between those who were foreign-born or not (Bourque et al., 2011; Selten et al., 2020).

Since the populations of the two Dutch sites (Amsterdam, Gouda & Voorhout) comprised all of the seven subgroups, rates were directly standardized for age and sex using their combined populations as the standard population. First, we standardized the rates separately for the reference populations and for minorities (all in one category) and, second, for minorities categorized according to the region of origin.

In the next step, differences in incidence were analyzed using Poisson regression models. We compared incidence rates across minorities by modelling ethnic minority status as a factor and additionally tested to what degree any effect of ethnic minority status would differ between sites by including terms for interaction. These models included age, gender, ethnic minority status, site and the first-order interaction of (site × ethnic minority status), and a scale factor to deal with possible over-dispersion.

The output for each site is presented in two ways: as adjusted IRR_adj_ resulting from a comparison to Amsterdam (Comparison A), and as IRR_adj_ emerging from a comparison within each site (Comparison B). Thus, we compared the incidence for, e.g. the reference population in Barcelona to that for the reference population in Amsterdam, and, e.g. the incidence for Black Africans in London to that for migrants from sub-Saharan Africa in Amsterdam (Comparison A). The choice for Amsterdam as reference was driven by the same reason as the selection of the combined populations of Gouda, Voorhout and Amsterdam to standardize rates: the presence of all seven sub-groups.

We also compared the incidence for minorities to that for the reference population at each site (Comparison B).

The terms for interaction associated with Comparison A indicate whether the variation between sites in the incidence among minorities is different from the variation between sites in the incidence among those of the reference population. The terms for interaction associated with Comparison B indicate whether there are differences between sites in the IRRs_adj_ among minorities compared to the reference population. The tests for the presence of interaction associated with both comparisons are identical. The IRRs_adj_ were also estimated for minorities broken down by region of origin. That is, the rate in a certain minority was compared to that in the reference population in a separate analysis with similar first-order interaction terms.

Data preparation, record linkage and estimation of standardized rates were performed using SPSS version 22.0. The Poisson regression analysis was conducted using STATA version 13.1.

## Results

### Number of cases and standardized rates

The analyses include 1886 cases with any psychotic disorder, 775 (41.4%) from minorities and 1111 (58.9%) from the reference populations.

The numbers of cases of NAPD among minorities and reference populations were 664 and 847, respectively. The remaining cases were diagnosed with APD (106 from minorities and 248 from reference populations) or could not be diagnosed with either APD or NAPD.

Of the 1886 included cases, 659 (34.9%) were interviewed. Relevant information on the remaining 1227 was retrieved from clinical notes or other sources. The proportions of individuals from minorities and reference populations who could be interviewed were 40.1 and 27.5%, respectively. These figures varied across sites and ranged from 21.9% *v.* 14.7% in London to 85.1% *v.* 77.8% in Valencia. In Amsterdam (36.0% *v.* 35.1%) and Val-de-Marne (26.2% *v.* 26.8%) these differences were minimal.

**Fig. 1.**
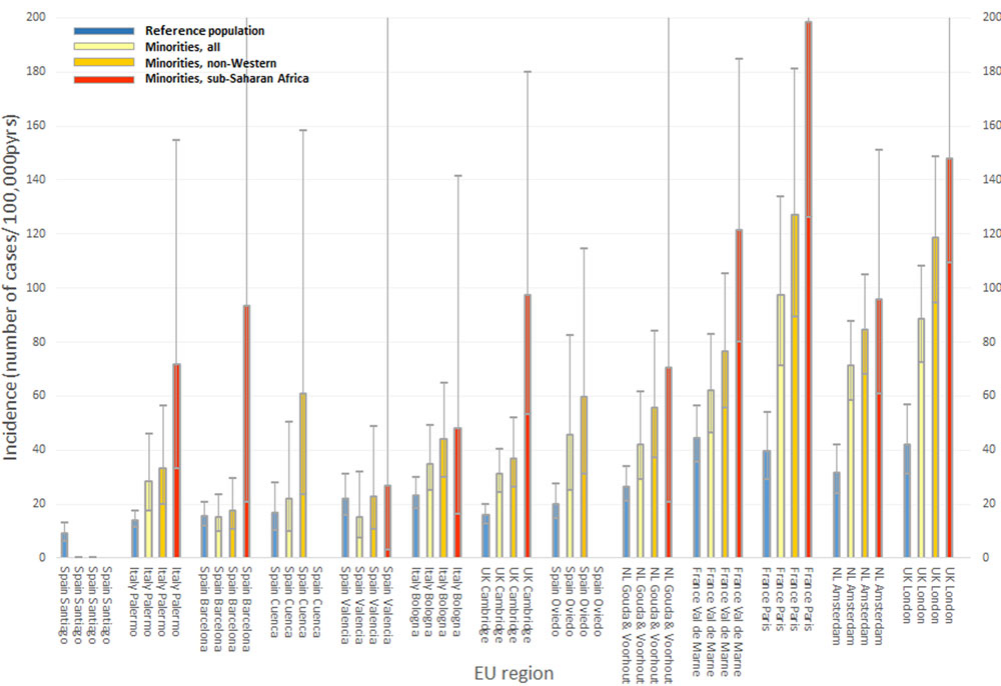
Age-and gender-adjusted Incidence rates (95% CI) of any psychotic disorder, by site and region of origin. Rates were calculated by combining the appropriate parameter estimates from the age- and gender-adjusted multivariable Poisson regression models including terms for the interaction of (site x ethnic minority status). (1) Reference population = individuals not belonging to a migrant or minority ethnic group. (2) Minorities, non-western  = individuals from the Middle-East, the Maghreb, sub-Saharan Africa, Asia, the Caribbean or Latin-America.

Table 1 shows the standardized incidence rates for each site. Figure 1 shows the rates (and 95% CI) calculated by combining the appropriate parameter estimates from the age- and gender-adjusted multivariable Poisson regression models (including terms for interaction). As reported previously (Jongsma et al., 2018), there were large differences in adjusted rates between the sites. We observed the highest rates of any psychosis among minorities and reference populations in the highly urbanized regions of London, Amsterdam, Paris, and Val-de-Marne (Table 1). The corresponding rates in Barcelona and Valencia were low.

**Table 1.** Numbers of cases and standardized incidence rates (*N* /100000 person-years) of any psychotic disorder, for reference populations and for migrant and minority ethnic groups (‘minorities’), 2010–2015

	Reference population		Minorities, all		Minorities, Western countries^a^		Minorities, Non-Western countries											
							Middle East^b^		The Maghreb^c^		sub-Saharan Africa		Asia^d^		The Caribbean^e^		Latin America	
	*N*	*Rate*^f^	*N*	*Rate*	*N*	*Rate*	*N*	*Rate*	*N*	*Rate*	*N*	*Rate*	*N*	*Rate*	*N*	*Rate*	*N*	*Rate*
London	64	*36.7*	198	*76.0*	29	*40.3*	10	*269.7*			67	*105.7*	21	*56.5*	43	*110.9*		
Cambridge	149	*12.0*	92	*24.5*	40	*21.7*	1	*9.4*			13	*73.0*	25	*15.9*	7	*47.4*		
Amsterdam	75	*24.9*	194	*58.3*	36	*40.3*	13	*51.8*	29	*102.7*	25	*78.4*	17	*35.3*	63	*64.8*	11	*88.4*
Gouda & Voorhout	126	*20.0*	38	*35.0*	8	*21.3*	2	*23.7*	18	*77.7*	3	*41.5*	3	*17.0*	1	*10.5*	3	*121.2*
Barcelona	74	*11.9*	26	*12.6*	8	*9.5*			4	*56.2*	2	*45.8*	3	*5.2*	0	*0.0*	9	*15.1*
Valencia	47	*16.8*	9	*12.2*	1	*3.6*			1	*34.9*	1	*17.0*	0	*0.0*	0	*0.0*	6	*26.7*
Oviedo	59	*15.0*	14	*44.7*	3	*21.6*			2	*66.1*	0	*0.0*	1	*59.7*	0	*0.0*	8	*69.0*
Santiago	36	*6.5*	0	*0.0*	0	*0.0*			0	*0.0*	0	*0.0*	0	*0.0*	0	*0.0*	0	*0.0*
Cuenca	20	*12.8*	7	*17.8*	2	*8.1*			2	*31.2*	0	*0.0*	0	*0.0*	0	*0.0*	3	*55.8*
Paris	60	*29.7*	57	*82.5*	4	*26.1*			13	*75.5*	25	*150.7*			4	*72.3*		
Val de Marne	130	*34.1*	71	*52.5*	4	*11.3*			17	*68.2*	30	*91.1*			7	*34.2*		
Bologna	113	*18.7*	48	*30.0*	14	*18.9*	1	*8.1*	8	*86.8*	4	*31.2*	17	*31.9*			4	*41.5*
Palermo	158	*10.5*	21	*27.3*	4	*18.0*	0	*0.0*	1	*23.4*	8	*45.4*	7	*15.9*			1	*41.4*
Total	1111		775^g^		153		27		95		178		94		125		45	

a Sites in Spain, Italy, Netherlands: Minorities from Europe, USA, Canada, Australia, New Zealand and countries of former Soviet Union; France: Minorities from Europe or Turkey; UK: self-identified Irish Whites or White Others.

b Turkey, Israel, Egypt, Iran, Iraq and other countries in the region; UK: self-identified Arabs.

c North-African countries, except Egypt.

d Including states of the former Soviet Union with a predominantly Islamic population.

e Caribbean islands, Surinam, Guyana, French Guyana and other French overseas departments.

f standardized incidence rates, N per 100,000 person-years.

g Including *N* = 58 subjects with ethnic background or region of origin that could not be categorized in one of the regions of origin shown in the Table.

With the exception of Santiago and Valencia, the incidence was higher among minorities than among reference populations. The rates for minorities showed a generally consistent pattern: increased for minorities in general, more increased for persons from non-Western countries and most increased for individuals from sub-Saharan Africa (Fig. 1). We obtained similar results when we restricted the analysis to NAPD (online Supplementary eTable S3 and eFig. S1).

### Recruitment sites compared to Amsterdam, for minorities (Comparison A)

Table 2 gives the results of a comparison of incidence rates for minorities at each site to that for minorities in Amsterdam, expressed as IRRs_adj_. The results show that the rate for minorities in Barcelona, combined into one category, was approximately five times lower than that in Amsterdam (IRR = 0.21, 95% CI 0.13–0.33), while the rate in Paris was higher than that in Amsterdam (IRR = 1.37, 95% CI 0.99–1.89). There were substantial differences between sites in incidence for minorities from Western countries and for those from non-Western countries combined into one category (Table 2), and for those from the Middle East, Asia, the Caribbean and Latin America (online Supplementary eTable S2). We obtained similar results for NAPD (data not shown).

**Table 2.** Age- and gender-adjusted Incidence rate ratios (IRR_adj_) of any psychotic disorder for reference populations and for different migrant and minority ethnic groups (‘minorities’), compared to the corresponding category in Amsterdam

	Reference population	Minorities, all	Minorities, Western countries^a^	Minorities, non-Western countries^b^
	IRR_adj_ (95% CI)	IRR_adj_ (95% CI)	IRR_adj_ (95%-CI)	IRR_adj_ (95% CI)
London	1.33 (0.92–1.92)	1.24 (0.99–1.54)	0.85 (0.51–1.44)	1.40 (1.10–1.79)
Cambridge	0.51 (0.37–0.69)	0.44 (0.33–0.57)	0.56 (0.35–0.91)	0.44 (0.31–0.62)
Amsterdam	1.00	1.00	1.00	1.00
Gouda & Voorhout	0.84 (0.62–1.16)	0.59 (0.40–0.87)	0.50 (0.22–1.13)	0.66 (0.43–1.01)
Barcelona	0.49 (0.34–0.70)	0.21 (0.13–0.33)	0.25 (0.11–0.57)	0.21 (0.12–0.35)
Valencia	0.70 (0.47–1.05)	0.22 (0.10–0.45)	0.09 (0.01–0.78)	0.27 (0.13–0.58)
Oviedo	0.64 (0.44–0.93)	0.64 (0.35–1.16)	0.54 (0.15–1.89)	0.71 (0.37–1.37)
Santiago	0.28 (0.18–0.44)	0.0 (–)	0.0 (–)	0.0 (–)
Cuenca	0.54 (0.31–0.92)	0.31 (0.13–0.71)	0.18 (0.04–0.82)	0.72 (0.28–1.89)
Paris	1.25 (0.86–1.82)	1.37 (0.99–1.89)	0.76 (0.25–2.28)	1.51 (1.04–2.17)
Val de Marne	1.42 (1.03–1.94)	0.87 (0.64–1.17)	0.47 (0.16–1.41)	0.91 (0.65–1.26)
Bologna	0.74 (0.53–1.02)	0.49 (0.35–0.69)	0.52 (0.27–1.00)	0.52 (0.35–0.78)
Palermo	0.44 (0.33–0.60)	0.40 (0.24–0.65)	0.40 (0.13–1.22)	0.39 (0.23–0.68)
Comparison between sites^c^	174⋅2/ 12/ <0.001	157⋅8/ 12/ <0.001	23.5/ 12/ 0.0235	120/ 12/ <0.001

a Including Europe, USA, Canada, USA, Australia, New Zealand and countries of the former Soviet Union, except states in Asia with a predominantly Islamic population.

b Middle East, The Maghreb, sub-Saharan Africa, Asia, The Caribbean (including French oversees departments), Latin America.

c Wald χ^2^ statistic/ df/ *p* value.

### Minorities compared to the reference population, by recruitment site (Comparison B)

Using the same Poisson model as in Comparison A, we estimated the IRRs_adj_ of any psychotic disorder for minorities compared with the local reference population at each site. The first column of Table 3 shows the results for all minorities combined. The differences in IRRs_adj_ between sites ranged from 0.70 in Valencia to 2.47 in Paris [test of interaction of (host region × ethnic minority status): χ^2^ = 22.6, *p* = 0.031]. Combining all sites, the mean IRR_adj_ of any psychotic disorder for all minorities was 2.37 (95% CI 2.06–2.73), after adjustment for age and gender, and 1.75 (95% CI 1.56–2.96) after adjustment for age, gender and site. Strongly overlapping 95% CIs were found for the IRRs_adj_ concerning first- and second-generation migrants in the Netherlands and in the UK, and no interaction of (site × generation) (Table 3). Importantly, at each site, the IRRs_adj_ for individuals from non-Western countries were higher than those for all minorities combined (Table 3). Combining all sites, the average IRR_adj_ of any psychotic disorder for individuals from non-Western countries, adjusted for age, gender and site, was 2.12 (95% CI 1.88–2.40). This figure was higher for those from sub-Saharan African countries: 3.23 (95% CI 2.66–3.93). Table 4 gives IRRs_adj_ for non-Western minorities. The IRRs_adj_ for Arabs in London (8.33, 95% CI 4.65–14.91) and for those from sub-Saharan African countries in Cambridge (5.55, 95% CI 3.04–10.12) were very high. We obtained similar results for NAPD (see online Supplementary eTable S4 and S5).

**Table 3.** Age- and gender-adjusted Incidence rate ratios (IRR_adj_) of any psychotic disorder for migrant and minority ethnic groups (‘minorities’), compared to the local reference population, by the site of recruitment

	Minorities, all	Minorities, Western countries^b^	Minorities, non-Western countries^a^	Minorities, all, by generation	
				first generation	Second generation (and higher)
	IRR_adj_ (95% CI)	IRR_adj_ (95% CI)	IRR_adj_ (95% CI)	IRR_adj_ (95% CI)	IRR_adj_ (95% CI)
London	2.11 (1.55–2.88)	0.97 (0.61–1.55)	2.73 (1.98–3.75)	2.03 (1.44–2.84)	2.32 (1.57–3.43)
Cambridge	1.95 (1.47–2.60)	1.67 (1.15–2.42)	2.20 (1.54–3.15)	1.86 (1.34–2.58)	2.58 (1.58–4.22)
Amsterdam	2.27 (1.69–3.04)	1.50 (0.98–2.29)	2.57 (1.91–3.45)	2.57 (1.86–3.54)	1.79 (1.21–2.64)
Gouda & Voorhout	1.58 (1.06–2.36)	0.89 (0.42–1.90)	1.99 (1.30–3.06)	1.95 (1.20–3.17)	1.19 (0.63–2.28)
Barcelona	0.97 (0.59–1.59)	0.76 (0.35–1.66)	1.08 (0.62–1.89)		
Valencia	0.70 (0.32–1.53)	0.20 (0.02–1.65)	0.99 (0.44–2.22)		
Oviedo	2.27 (1.19–4.31)	1.26 (0.37–4.34)	2.83 (1.41–5.67)		
Santiago	0.00 (–)	0.0 (–)	0.00 (–)		
Cuenca	1.30 (0.50–3.37)	0.50 (0.11–2.37)	3.44 (1.20–9.90)		
Paris	2.47 (1.66–3.69)	0.91 (0.31–2.68)	3.09 (2.02–4.73)		
Val de Marne	1.39 (1.01–1.91)	0.50 (0.17–1.43)	1.64 (1.16–2.31)		
Bologna	1.50 (1.04–2.18)	1.05 (0.58–1.90)	1.80 (1.19–2.72)		
Palermo	2.02 (1.22–3.34)	1.37 (0.48–3.93)	2.27 (1.32–3.89)		
Interaction of	22.6/ 12/ 0.031^c^	13.9/ 12/ 0.3062	20.3/ 12/ 0.0611	Interaction of	9.2/ 6/ 0.1652
(site × minority status)				(site x generation)	
Pooled IRR_adj_^d^	1.75 (1.56–1.96)	1.09 (0.91–1.32)	2.12 (1.88–2.40)		

a Including Europe, USA, Canada, USA, Australia, New Zealand and countries of former Soviet Union, except states in Asia with a predominantly Islamic population.

b Midde East, The Maghreb, sub-Saharan Africa, Asia, The Caribbean (including French oversees departments), Latin America.

c Wald χ^2^ statistic/ df/*p* value.

d adjusted for age, gender and recruitment site.

**Table 4.** Age- and gender-adjusted Incidence rate ratios (IRR_adj_) of any psychotic disorder, for certain non-Western migrant and minority ethnic groups (‘minorities’), compared to the local reference population, by the site of recruitment

	Minorities from:					
	Middle East^a^	The Maghreb^b^	sub-Saharan Africa	Asia^c^	The Caribbean^d^	Latin America
	IRR_adj_ (95% CI)	IRR_adj_ (95% CI)	IRR_adj_ (95% CI)	IRR_adj_ (95% CI)	IRR_adj_ (95% CI)	IRR_adj_ (95% CI)
London	8.33 (4.65–14.91)		3.26 (2.27–4.69)	1.21 (0.71–2.07)	3.32 (2.23–4.92)	
Cambridge	1.38 (0.25–7.66)		5.55 (3.04–10.12)	1.54 (0.97–2.43)	4.03 (1.86–8.75)	
Amsterdam	1.96 (1.17–3.27)	3.65 (2.33–5.72)	3.15 (1.95–5.09)	1.49 (0.84–2.63)	2.58 (1.83–3.64)	3.20 (1.59–6.43)
Gouda & Voorhout	1.00 (0.30–3.39)	3.27 (1.95–5.49)	2.41 (0.72–8.10)	0.75 (0.22–2.61)	0.56 (0.07–4.16)	4.98 (1.41–17.55)
Barcelona		2.57 (1.01–6.55)	2.64 (0.78–8.90)	0.38 (0.11–1.32)	0.00 (–)	0.98 (0.46–2.10)
Valencia		↑^e^	↑	↑	↑	1.35 (0.53–3.45)
Oviedo		4.36 (1.53–12.39)	0.00 (–)	2.43 (0.29–20.64)	↑	2.96 (1.31–6.68)
Santiago		↑	↑	↑	↑	0.00 (–)
Cuenca		↑	↑	↑	↑	3.80 (1.00–14.45)
Paris		1.98 (1.06–3.72)	4.61 (2.81–7.56)		2.83 (1.01–7.97)	
Val de Marne		1.27 (0.74–2.15)	2.48 (1.63–3.79)		0.97 (0.45–2.12)	
Bologna	1.00 (0.18–5.57)	2.76 (1.30–5.86)	1.87 (0.65–5.39)	1.49 (0.86–2.60)		2.35 (0.78–7.05)
Palermo	0.00 (–)	0.96 (0.12–7.49)	4.64 (2.18–9.85)	1.70 (0.75–3.87)		1.83 (0.21–15.91)
Interaction of	20.0/ 5/ 0.0013^f^	12.5/ 7/ 0.0849	8.6/ 9/ 0.4713	6.1/ 7/ 0.5283	11.1/ 6/ 0.0852	9.3/ 8/ 0.3185
(site × minority status						
Pooled IRR_adj_^g^	2.34 (1.61–3.41)	2.39 (1.88–3.02)	3.23 (2.66–3.93)	1.28 (1.00–1.64)	2.38 (1.92–2.95)	1.92 (1.31–2.81)

a Turkey, Israel, Egypt, Iran, Iraq and other countries in the region; UK: self-identified Arabs.

b North-African countries, except Egypt.

c Including those states of the former Soviet Union with a predominant Islamic population.

d Surinam, Guyana, French Guyana and the other French overseas departments.

e ↑ site collapsed with the preceding one.

f Wald χ^2^ statistic/ df/*p* value.

g adjusted for age, gender and recruitment site.

## Discussion

We observed large between-site differences in incidence rates of psychotic disorder among minorities from the same region of origin. On average, minorities had a rate of psychotic disorder which was almost double that of the reference population, although this ratio varied between settings, from 0.70 to 2.47. A gradient in the IRRs_adj_ (minorities *v.* reference population), that is, a higher IRR_adj_ for subjects from non-Western countries than for those from Western countries and the highest IRR_adj_ for those of sub-Saharan African origin, was found at most sites. IRRs_adj_ were also very high for North-Africans in The Netherlands and for African-Caribbeans in the UK. IRRs_adj_ were lower for minorities in the highly urbanized Spanish cities than in London, Paris or Amsterdam.

### Limitations

This is the first study to compare psychosis incidence rates among minorities across several countries using uniform methodologies (Jongsma et al., 2018). Several limitations need to be considered.

First, at some sites the numbers of cases among minorities were small and the confidence intervals for the results correspondingly large. However, at each site the size of denominator data for migrants was sufficient to predict at least one new case from a minority based on the incidence rate of the reference population. The more striking is the consistent pattern across sites in Fig. 1 with higher risks among those of non-Western origin and even higher risk for those from sub-Saharan Africa. So, the Figure should be understood in terms of this consistency and not in terms of statistical significance of specific comparisons.

Second, there are differences between regions in mental health care systems, which may result in differential barriers to care. This could influence the probability of mental health care utilization, especially among individuals who face additional linguistic and cultural barriers (Lindert, Schouler-Ocak, Heinz, & Priebe, 2008). Of note, the duration of untreated psychosis (DUP) (the interval between onset and treatment) might be an indicator of the accessibility of mental health services. However, a review of studies investigating the association between migrant or ethnic minority status and DUP did not find evidence for longer DUPs or treatment delays (Anderson, Flora, Archie, Morgan, & McKenzie, 2014). Importantly, the onset of psychotic symptoms is a serious event with a very high chance to result in contact with the mental health care system (Link & Dohrenwend, 1980; Prince & Phelan, 1994; Von Korff et al., 1985). Furthermore, if thresholds for service contact are higher for minorities, our estimated IRRs_adj_ are underestimates.

Third, individuals were sampled using a first-contact design. Hogerzeil, van Hemert, Veling, and Hoek (2017) compared such a design to that of a longitudinal psychiatric registry and found that the first-contact method underestimated the risk for the reference population due to the tendency to miss older patients who had been treated for another psychiatric disorder prior to the onset of a psychotic disorder. Nonetheless, using the longitudinal registry method they still observed increased relative risks for minorities (Hogerzeil et al., 2017). Fourth, the incidence among minorities may be underestimated. Some first-generation migrants may not be included as incident due to salmon bias, which arises when migrants return to their home country to seek treatment. Furthermore, studies found that a lower socioeconomic status was associated with a higher ratio of untreated to treated cases of major mental disorder (Link & Dohrenwend, 1980). Fifth, we encountered some challenges with denominator data in France, Italy and Spain where one cannot distinguish between second-generation migrants and the native-born population. This may have artificially reduced the IRRs_adj_ among first-generation migrants in France, but is unlikely to have influenced the results for Italy and Spain where individuals of the second generation were small in number and almost exclusively under the age of 18 at the time of the study (Bonifazi, 2000; Reher & Requena, 2009). Further, this is unlikely to explain the variation in IRRs_adj_ between sites, because this was mainly driven by the difference between Spanish and non-Spanish sites.

Sixth, the denominator data were stratified by age, gender, migrant or ethnic minority group, and recruitment site. Thus, this incidence study was restricted to these variables and important determinants such as socio-economic disadvantage or cannabis use could not be taken into account. Analyses of the EU-GEI case-control data will enable the examination of the impact of these factors on the risk of psychosis among minorities and on regional differences in rates and rate ratios.

Seventh, we used two different definitions for the minority: one based on self-assigned ethnicity (for the UK) and another on migration history. Since the large ethnic minority groups in Europe do have a recent migration history, most members are also first – or second-generation migrants. As the denominator data (from the census) in the UK use a combination of self-assigned ethnic minority and birth country (that is, UK or abroad), we used a definition for the UK that was different from the definition used in the other countries. This was done to make a valid estimation of the IRRs for the UK possible, as the definition for the cases and the denominator should always match. The results do not suggest that the data of the UK have an outlier position that may have been caused by a slightly different definition.

Finally, the aggregation of minorities from similar regions may mask within-group heterogeneity. For instance, individuals from the Middle East are classified as one group but represent a population with substantial variation in language, culture and socioeconomic position.

### Findings in context

Our findings are consistent with a recent meta-analysis showing 2- to 3-fold increases in the relative risks of first- and second-generation migrants in Europe (Selten et al., 2020). The important caveat to this is that there was substantial variation, which means overall rate ratios can be misleading. We also found the highest relative risks among individuals from non-Western countries, in particular those from Africa and the Caribbean (Dykxhoorn et al., 2019; Selten et al., 2020). Our finding of comparatively high relative risks of psychotic disorder for minorities in less urbanized environments is consistent with evidence from rural UK (Kirkbride et al., 2017).

### Interpretation of findings

The substantial inter-site differences in the incidence rates for minorities and the marked variation in the IRRs_adj_ indicate that their risk is not a fixed quantity independent from the environment. Consequently, the implications of our findings reach beyond the topic of migration and psychosis. Importantly, there is no evidence that the high incidence among individuals from non-Western countries in Europe reflects a similarly high incidence in the country of origin. While there have been no high-quality incidence studies from Africa, incidence studies from the Caribbean (Bhugra et al., 1996; Hickling & Rodgers-Johnson, 1995; Mahy, Mallett, Leff, & Bhugra, 1999) and Surinam (Hanoeman, Selten, & Kahn, 2002; Selten et al., 2005) and prevalence studies from India and China (Baxter et al., 2016) have reported rates within the range reported for other populations worldwide.

Several explanations have been forwarded to explain the increased incidence among minorities, but a definitive explanation is lacking (Cantor-Graae & Selten, 2005). A socio-developmental model posits that greater exposure to social risk over the life course, particularly those involving threat, hostility and violence, explains some of the high rates in various minorities (Morgan, Knowles, & Hutchinson, 2019). The social defeat hypothesis of psychosis proposes that an inferior position or an outsider status leads to increased baseline activity and/or sensitization of the mesolimbic dopamine system, placing an individual at an increased risk for psychotic disorder (Gevonden et al., 2014; Selten & Cantor-Graae, 2005; Selten, Booij, Buwalda, & Meyer-Lindenberg, 2017; Selten, van der Ven, Rutten, & Cantor-Graae, 2013). Egerton et al. (2017) supported this hypothesis by reporting an elevated striatal dopamine function in migrants and their children, but this study awaits replication in a larger sample. The British epidemiologist Marmot observed that persons lower in the hierarchy are more likely to be affected by a wide range of diseases, a phenomenon which he coined status syndrome (Marmot, 2015). He argued that key factors related to a person's position in the hierarchy include a subjective sense of control over one's life (autonomy) and the opportunity for social participation. Our findings are in line with his ideas.

Of note, the researchers of the present study also collected information on 1497 population-based controls at the pertinent sites. A recent analysis of this data showed that the adjusted odds ratio (OR) of psychosis for ethnic minorities compared to the white majority (1.61, 95% CI 1.31–1.98) decreased following adjustment for social disadvantage (1.52, 95% CI 1.22–1.98) and self-reported fluency in the dominant language (1.22, 95% CI 0.95–1.57) (Jongsma et al., 2020). Indeed, an imperfect mastery of the dominant language may contribute to an experience of social disempowerment and social defeat.

Future studies of the EU-GEI case-control data will evaluate what factors explain the regional differences reported here. This may ultimately lead to tailored and site-specific interventions and may also give insight into protective factors that help persons from minority populations to cope with exposure to high risk. Future studies could also examine trends in incidence rates across calendar time to find out whether the high risks among ethnic minorities persist or decline following specific interventions. Given the dearth of knowledge about incidence or prevalence in North- and sub-Saharan Africa, epidemiological studies in these regions would also be very useful.

## Conclusions

Although we found a 7-fold variation in the incidence rates of psychosis among minorities, a consistent gradient of rates (reference population < members of any minority < individuals from non-Western countries < individuals from sub-Saharan Africa) emerged across sites. Our findings highlight the significance of the social context for the etiology of the disorder.

## Supplementary material

For supplementary material accompanying this paper visit https://doi.org/10.1017/S0033291720003219. click here to view supplementary material
