# Supplementary material for: The incidence of psychotic disorders among migrants and minority ethnic groups in Europe: findings from the multinational EU-GEI study
Source: Psychol Med. 2020 Sep 22;52(7):1376–85. doi: 10.1017/S0033291720003219 (PMC9157293; doi:10.1017/S0033291720003219)
Supplement: Supplementary file 1 [file S0033291720003219sup001.docx]

**Supplement eMethods, eTables and eFigure**

**Supplement eMethods**

**Case ascertainment**

eTable 1. Denominator by study site for reference populations and for migrant and minority ethnic groups (“minorities”), overall and by region of origin.

eTable 2. Age- and gender-adjusted Incidence rate ratios (IRR_adj_) of any psychotic disorder for certain non-Western migrant and minority ethnic groups (“minorities”), compared to the corresponding category in Amsterdam (see Table 2.).

eTable 3. Numbers of cases and standardized incidence rates (N/ 100,000 person-years) of NAPD, for reference populations and for migrant and minority ethnic groups (“minorities”), 2010-2015.

eFigure 1. Age-and gender-adjusted Incidence rates (95%-CI) of NAPD, by site and region of origin. See also Figure 1 in main text.

eTable 4. Age- and gender-adjusted Incidence rate ratios (IRR_adj_) of NAPD for migrant and minority ethnic groups (“minorities”), compared to the local reference population, by site of recruitment.

eTable 5. Age- and gender-adjusted Incidence rate ratios (IRR_adj_) of NAPD, for certain non-Western migrant and minority ethnic groups (“minorities”), compared to the local reference population, by site of recruitment.

**Supplement eMethods**

**Case ascertainment**

Trained researchers who were part of a local research team overseen by a psychiatrist with experience in epidemiology, regularly contacted clinicians to identify suspected cases of first-episode psychosis (FEP) in circumscribed catchment areas. Researchers received training in diagnostic instruments and epidemiological principles. In case of doubt about whether a person concerned a true incident case, consensus within the research team was met. The inclusion criteria for this study were age 18 to 64 years, residing in the catchment area at first identification and an International Statistical Classification of Diseases and Related Health Problems, Tenth Revision (ICD-10) diagnosis of FEP (codes F20-F33) that had never been treated. Individuals with psychotic symptoms precipitated by an organic cause or transient psychotic symptoms resulting from acute intoxication (*ICD-10* code F1X.5) were excluded. In order to ensure comparability of diagnoses across catchment areas, we obtained diagnoses derived from the Operational Criteria Checklist algorithm (OPCRIT). The information for OPCRIT assessment was obtained from a semi-structured clinical interview or, if cases refused the interview, reconstructed based on clinical notes and other relevant information. If OPCRIT assessment could not be completed, we recorded the clinical diagnosis. All assessors were extensively trained in OPCRIT and reached acceptable inter-rater agreement (κ = 0·7). The clinical interview schedule used at each site followed local expertise, including the Schedules for Clinical Assessment in Neuropsychiatry ("World Health Organization. Schedules for Clinical Assessment in Neuropsychiatry," 1992) (United Kingdom and Italy), the Comprehensive Assessment of Symptoms and History (Andreasen, Flaum, & Arndt, 1992) (the Netherlands), and the Diagnostic Interview for Genetic Studies (Nurnberger et al., 1994) (France).

**Rationale for exclusion of four sites**

We excluded the catchment areas Puy-de-Dôme (France) and Madrid (Spain) because of missing information on country of birth or ethnicity for many cases. Ribeirão Preto (Brazil) was excluded because of missing information on country of birth and a classification of ethnicity based on skin colour without information concerning region of origin. Verona (Italy) was excluded owing to missing information on country of birth in the denominator.

**Population at risk**

**France categories of birth country**

It is important to note that the French bureau distinguishes only seven categories: (1) mainland France; (2) the European Union; (3) other European countries and Turkey; (4) The Maghreb; (5) sub-Saharan African countries; (6) overseas departments: French Guyana (South-America), the islands of Guadeloupe and Martinique (Caribbean region), Réunion and Mayotte (islands near Madagascar), New Caledonia and French Polynesia (in Pacific Ocean); (7) Other countries.

**UK Census categories**

For the catchment areas in the UK we used data from the 2011 census, which recognizes the following categories, based on self-assigned ethnicity and country of birth (UK or not): White British, White Irish, White traveller or gypsy, Other White, mixed White Black- Caribbean, mixed White Black-African, mixed White Asian, mixed Other, Indian, Pakistani, Bangladeshi, Chinese, Other Asian, Black Caribbean, Black African, Other Black, Arab, Other and unknown. We compared the risk for ethnic minorities in the UK to that for the White British.

**Exposure classification**

To enable a cross-site comparison including the UK, we compared the rate of psychosis for migrants from (i) Western countries in Italy, Spain and the Netherlands to that for the White Irish and Other Whites in the UK and to that for migrants from European countries or Turkey in France; (ii) Sub-Saharan Africa in continental Europe to that for Black Africans and Mixed White-Black-Africans in the UK; (iii) (a) the Netherlands Antilles (Caribbean islands) or Surinam in the Netherlands, (b) Black Caribbeans and Mixed White-Black Caribbeans in the UK, and (c) migrants from Overseas Departments in France; (iv) Asia (except the Middle East) in Italy, Spain and the Netherlands to that for Indians, Pakistani, Bangladeshi, Chinese, Other Asians (non-Arab) and mixed White-Asians in the UK; (v) the Middle-East in Italy and The Netherlands to that for Arabs in the UK; (vi) non-Western countries in Italy, Spain and the Netherlands to that for non-whites in the UK, and to that for migrants from categories 4 through 7 in France.

**eTable 1. Denominator by study site for reference populations and for migrant and minority ethnic groups (“minorities”), overall and by region of origin.**

|  | **Reference population** | **Minorities, all** |  | **Minorities, Western countries ^1)^** |  |  |  |  |  |  |  |  | **Unclassifiable** |
| --- | --- | --- | --- | --- | --- | --- | --- | --- | --- | --- | --- | --- | --- |
|  |  |  |  |  |  | **Middle East ^2)^** | **The Maghreb**  **^3)^** | **Sub-Saharan Africa** | **Asia ^4)^** | **The Caribbean ^5)^** | **Latin America** |  |  |
|  |  |  |  |  |  |  |  |  |  |  |  |  |  |
| London | 175,706 | 250,747 |  | 80,459 |  | 3,054 |  | 57,863 | 41,264 | 37,070 |  |  | 31,037 |
| Cambridge | 1,238,172 | 316,251 |  | 163,242 |  | 4,101 |  | 15,369 | 104,043 | 11,976 |  |  | 17,520 |
|  |  |  |  |  |  |  |  |  |  |  |  |  |  |
| Amsterdam | 295,587 | 330,084 |  | 87,219 |  | 24,786 | 29,049 | 32,628 | 46,752 | 97,167 | 12,483 |  |  |
| Gouda & Voorhout | 651,786 | 114,984 |  | 44,418 |  | 8,526 | 22,395 | 4,899 | 23,784 | 8,310 | 2,652 |  |  |
|  |  |  |  |  |  |  |  |  |  |  |  |  |  |
| Barcelona | 688,283 | 195,603 |  | 77,304 |  |  | 7,607 | 2,179 | 37,838 | 4,583 | 66,090 |  |  |
| Valencia | 299,983 | 64,206 |  | 24,141 |  |  | 4,005 | 4,268 | 8,389 | 1,067 | 22,332 |  |  |
| Oviedo | 428,483 | 34,141 |  | 13,524 |  |  | 2,073 | 2,522 | 1,349 | 383 | 14,288 |  |  |
| Santiago | 556,191 | 18,724 |  | 7,062 |  |  | 1,375 | 582 | 931 | 198 | 8,573 |  |  |
| Cuenca | 160,724 | 34,348 |  | 25,139 |  |  | 3,093 | 197 | 729 | 159 | 5,030 |  |  |
|  |  |  |  |  |  |  |  |  |  |  |  |  |  |
| Paris | 179,220 | 89,143 |  | 16,859 |  |  | 29,531 | 20,232 |  | 5,292 |  |  | 17,227 |
| Val de Marne | 342,091 | 168,542 |  | 28,923 |  |  | 49,875 | 36,860 |  | 21,104 |  |  | 31,778 |
|  |  |  |  |  |  |  |  |  |  |  |  |  |  |
| Bologna | 727,700 | 172,224 |  | 77,354 |  | 4,505 | 14,880 | 10,191 | 56,311 |  | 8,973 |  |  |
| Palermo | 1,493,856 | 100,863 |  | 30,793 |  | 751 | 11,404 | 17,069 | 35,560 |  | 5,274 |  |  |
|  |  |  |  |  |  |  |  |  |  |  |  |  |  |

^1)^ Sites in Spain, Italy, Netherlands: Minorities from Europe, USA, Canada, Australia, New Zealand and countries of former Soviet Union; France: Minorities from Europe or Turkey; UK: self-identified Irish Whites or White Others; ^2)^ Turkey, Israel, Egypt, Iran, Iraq and other countries in the region; UK: self-identified Arabs ; ^3)^ North-African countries, except Egypt; ^4)^ including states of the former Soviet Union with a predominantly Islamic population; ^5)^ Caribbean islands, Surinam, Guyana, French Guyana and other French overseas departments

**eTable 2. Age- and gender-adjusted Incidence rate ratios (IRR_adj_) of any psychotic disorder for certain non-Western migrant and minority ethnic groups (“minorities”), compared to the corresponding category in Amsterdam (see Table 2.).**

|  | **Minorities from:** |  |  |  |  |  |  |
| --- | --- | --- | --- | --- | --- | --- | --- |
|  | **Middle East^1)^** | **The Maghreb^2)^** | **sub-Saharan Africa** | **Asia^3)^** | **The Caribbean^4)^** | **Latin America** |  |
|  | **IRR_adj_ [95%-CI]** | **IRR_adj_ [95%-CI]** | **IRR_adj_ [95%-CI]** | **IRR_adj_ [95%-CI]** | **IRR_adj_ [95%-CI]** | **IRR_adj_ [95%-CI]** |  |
|  |  |  |  |  |  |  |  |
| London | 5·58 [2·72-11·47] |  | 1·36 [0·84-2·22] | 1·07 [0·54-2·15] | 1·71 [1·15-2·55] |  |  |
| Cambridge | 0·36 [0·06-2·13] |  | 0·90 [0·44-1·83] | 0·53 [0·27-1·03] | 0·79 [0·35-1·75] |  |  |
|  |  |  |  |  |  |  |  |
| Amsterdam | 1·00 | 1·00 | 1·00 | 1·00 | 1·00 | 1·00 |  |
| Gouda & Voorhout | 0·44 [0·12-1·60] | 0·77 [0·42-1·43] | 0·65 [0·18-2·31] | 0·43 [0·11-1·64] | 0·18 [0·02-1·36] | 1·33 [0·33-5·44] |  |
|  |  |  |  |  |  |  |  |
| Barcelona |  | 0·40 [0·15-1·08] | 0·47 [0·13-1·67] | 0·14 [0·04-0·55] | 0·00 [-] | 0·15 [0·06-0·40] |  |
| Valencia |  | ↑^6)^ | ↑ | ↑ | ↑ | 0·30 [0·10-0·89] |  |
| Oviedo |  | 0·54 [0·18-1·63] | 0·00 [-] | 0·74 [0·08-6·62] | ↑ | 0·59 [0·22-1·62] |  |
| Santiago |  | ↑ | ↑ | ↑ | ↑ | 0·0 [-] |  |
| Cuenca |  | ↑ | ↑ | ↑ | ↑ | 0·64 [0·16-2·62] |  |
|  |  |  |  |  |  |  |  |
| Paris |  | 0·68 [0·34-1·35] | 1·82 [1·01-3·28] |  | 1·37 [0·49-3·86] |  |  |
| Val de Marne |  | 0·49 [0·26-0·92] | 1·12 [0·64-1·96] |  | 0·53 [0·24-1·18] |  |  |
|  |  |  |  |  |  |  |  |
| Bologna | 0·38 [0·06-2·24] | 0·57 [0·25-1·30] | 0·44 [0·14-1·35] | 0·75 [0·36-1·56] |  | 0·55 [0·16-1·94] |  |
| Palermo | 0·00 [-] | 0·12 [0·01-0·95] | 0·66 [0·28-1·53] | 0·51 [0·20-1·33] |  | 0·26 [0·03-2·43] |  |
|  |  |  |  |  |  |  |  |
| Comparison between sites | 34·3/ 5/ 0·001^5)^ | 10·3/ 7/ 0·1736 | 16·1/ 9/ 0·0654 | 14·4/ 7/ 0·0446 | 16·1/ 6/ 0·0133 | 19·3/ 8/ 0·0132 |  |
|  |  |  |  |  |  |  |  |

^1)^ Turkey, Israel, Egypt, Iran, Iraq and other countries in the region; UK: self-identified Arabs; ^2)^North-African countries, except Egypt; ^3)^including those states of the former Soviet Union with a predominant Islamic population; ^4)^Surinam, Guyana, French Guyana and the other French overseas departments; ^5)^ Wald χ2 statistic/ df/ P value; ^6)^↑ site collapsed with the preceding one

**eTable 3. Numbers of cases and standardized incidence rates (N/ 100,000 person-years) of NAPD, for reference populations and for migrant and minority ethnic groups (“minorities”), 2010-2015.**

|  | **Reference population** | |  | **Minorities, all** | |  | **Minorities, Western countries ^1)^** | |  | **Minorities, Non-Western countries** | | | | | | | | | | | |
| --- | --- | --- | --- | --- | --- | --- | --- | --- | --- | --- | --- | --- | --- | --- | --- | --- | --- | --- | --- | --- | --- |
|  |  |  |  |  |  |  |  |  |  | Middle East ^2)^ | | The Maghreb  ^3)^ | | sub-Saharan Africa | | Asia ^4)^ | | The Caribbean ^5)^ | | Latin America | |
|  | N | *Rate* |  | N | *Rate* |  | N | *Rate* |  | N | *Rate* | N | *Rate* | N | *Rate* | N | *Rate* | N | *Rate* | N | *Rate* |
|  |  |  |  |  |  |  |  |  |  |  |  |  |  |  |  |  |  |  |  |  |  |
| London | 58 | 32·1 |  | 187 | 72·2 |  | 26 | *36·7* |  | 10 | 269·7 |  |  | 65 | 102·8 | 20 | 54·5 | 39 | 100·2 |  |  |
| Cambridge | 94 | 7·6 |  | 64 | 16·5 |  | 28 | *15·0* |  | 1 | 9·4 |  |  | 9 | 50·4 | 19 | 12·6 | 4 | 26·6 |  |  |
|  |  |  |  |  |  |  |  |  |  |  |  |  |  |  |  |  |  |  |  |  |  |
| Amsterdam | 58 | 19·4 |  | 179 | 53·8 |  | 34 | *38·1* |  | 11 | 43·9 | 26 | 92·3 | 24 | 75·1 | 15 | 31·5 | 58 | 59·7 | 11 | 88·4 |
| Gouda & Voorhout | 89 | 14·3 |  | 32 | 29·4 |  | 5 | *14·3* |  | 2 | 23·7 | 15 | 65·0 | 3 | 41·5 | 3 | 17·0 | 1 | 10·5 | 3 | 121·2 |
|  |  |  |  |  |  |  |  |  |  |  |  |  |  |  |  |  |  |  |  |  |  |
| Barcelona | 63 | 10·2 |  | 26 | 12·6 |  | 8 | *9·5* |  |  |  | 4 | 56·2 | 2 | 45·8 | 3 | 5·2 | 0 | 0·0 | 9 | 15·1 |
| Valencia | 42 | 14·8 |  | 7 | 10·2 |  | 1 | *3·6* |  |  |  | 1 | 34·9 | 0 | 0·0 | 0 | 0·0 | 0 | 0·0 | 5 | 23·7 |
| Oviedo | 42 | 10·2 |  | 12 | 35·6 |  | 3 | *21·6* |  |  |  | 2 | 66·1 | 0 | 0·0 | 1 | 59·7 | 0 | 0·0 | 6 | 50·4 |
| Santiago | 28 | 4·9 |  | 0 | 0·0 |  | 0 | *0·0* |  |  |  | 0 | 0·0 | 0 | 0·0 | 0 | 0·0 | 0 | 0·0 | 0 | 0·0 |
| Cuenca | 20 | 12·8 |  | 6 | 15·9 |  | 2 | *8·1* |  |  |  | 2 | 31·2 | 0 | 0·0 | 0 | 0·0 | 0 | 0·0 | 2 | 33·1 |
|  |  |  |  |  |  |  |  |  |  |  |  |  |  |  |  |  |  |  |  |  |  |
| Paris | 54 | 26·4 |  | 51 | 69·8 |  | 4 | *26·1* |  |  |  | 12 | 71·8 | 22 | 125·5 |  |  | 4 | 72·3 |  |  |
| Val de Marne | 86 | 22·4 |  | 42 | 32·1 |  | 1 | *5·0* |  |  |  | 9 | 37·0 | 20 | 63·1 |  |  | 4 | 19·9 |  |  |
|  |  |  |  |  |  |  |  |  |  |  |  |  |  |  |  |  |  |  |  |  |  |
| Bologna | 82 | 13·6 |  | 38 | 24·6 |  | 10 | *13·3* |  | 0 | 0·0 | 8 | 86·8 | 4 | 31·2 | 15 | 27·0 |  |  | 1 | 7·6 |
| Palermo | 131 | 8·7 |  | 20 | 25·6 |  | 4 | *18·0* |  | 0 | 0·0 | 1 | 23·4 | 8 | 45·4 | 6 | 13·0 |  |  | 1 | 41·4 |
|  |  |  |  |  |  |  |  |  |  |  |  |  |  |  |  |  |  |  |  |  |  |
| Total | 847 |  |  | 664^6)^ |  |  | 126 |  |  | 24 |  | 80 |  | 157 |  | 82 |  | 110 |  | 38 |  |
|  |  |  |  |  |  |  |  |  |  |  |  |  |  |  |  |  |  |  |  |  |  |

^1)^ Sites in Spain, Italy, Netherlands: Minorities from Europe, USA, Canada, Australia, New Zealand and countries of former Soviet Union; France: Minorities from Europe or Turkey; UK: self-identified Irish Whites or White Others; ^2)^ Turkey, Israel, Egypt, Iran, Iraq and other countries in the region; UK: self-identified Arabs ; ^3)^ North-African countries, except Egypt; ^4)^ including those states of the former Soviet Union with a predominantly Islamic population; ^5)^ Caribbean islands, Surinam, Guyana, French Guyana and other French overseas departments; ^6)^ Including N=47 subjects with ethnic background or region of origin that could not be categorized in one of the regions of origin shown in the Table

**eFigure 1. Age-and gender-adjusted Incidence rates (95%-CI) of NAPD, by site and region of origin. See also Figure 1 in main text.**


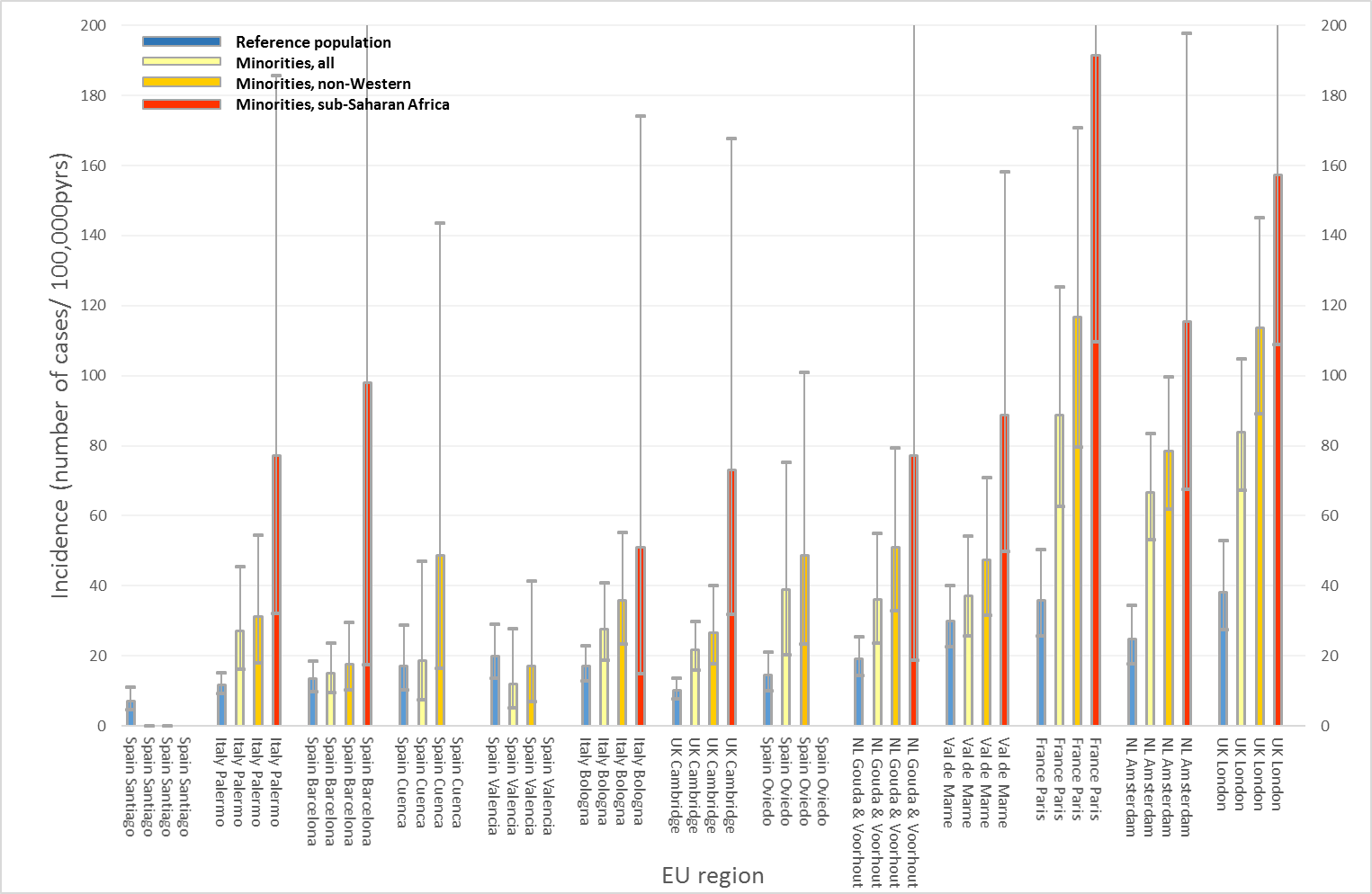


**Legend to eFigure 1**

^1)^ Reference population= individuals not belonging to a migrant or minority ethnic group.

^2)^ Individuals from Non-Western countries = individuals from the Middle-East, the Maghreb, sub-Saharan Africa, Asia, the Caribbean or Latin-America.

^3)^ Rates were calculated by combining the appropriate parameter estimates from the age- and gender-adjusted multivariable Poisson regression models (including terms for interaction).

^4)^ Rates were calculated by combining the appropriate parameter estimates from the age- and gender-adjusted multivariable Poisson regression models including terms for interaction of {site x ethnic minority status}.

**eTable 4. Age- and gender-adjusted Incidence rate ratios (IRR_adj_) of NAPD for migrant and minority ethnic groups (“minorities”), compared to the local reference population, by site of recruitment.**

|  | **Minorities, all** |  | **Minorities,** |  | **Minorities,** |  |  |
| --- | --- | --- | --- | --- | --- | --- | --- |
|  |  |  | **Western countries^1)^** |  | **non-Western countries^2)^** |  |  |
|  | IRR_adj_ [95%-CI] |  | IRR_adj_ [95%-CI] |  | IRR_adj_ [95%-CI] |  |  |
|  |  |  |  |  |  |  |  |
| London | 2·21 [1·58-3·08] |  | 0·95 [0·57-1·59] |  | 2·89 [2·06-4·05] |  |  |
| Cambridge | 2·13 [1·48-3·05] |  | 1·82 [1·14-2·90] |  | 2·48 [1·61-3·83] |  |  |
|  |  |  |  |  |  |  |  |
| Amsterdam | 2·70 [1·93-3·78] |  | 1·81 [1·13-2·90] |  | 3·05 [2·19-4·26] |  |  |
| Gouda & Voorhout | 1·88 [1·19-2·97] |  | 0·78 [0·29-2·12] |  | 2·53 [1·58-4·05] |  |  |
|  |  |  |  |  |  |  |  |
| Barcelona | 1·12 [0·67-1·88] |  | 0·87 [0·38-1·97] |  | 1·25 [0·70-2·21] |  |  |
| Valencia | 0·59 [0·24-1·47] |  | 0·22 [0·02-1·98] |  | 0·81 [0·32-2·07] |  |  |
| Oviedo | 2·69 [1·30-5·58] |  | 1·74 [0·48-6·40] |  | 3·21 [1·46-7·05] |  |  |
| Santiago | 0·00 [-] |  | 0·0 [-] |  | 0·00 [-] |  |  |
| Cuenca | 1·09 [0·39-3·08] |  | 0·49 [0·10-2·46] |  | 2·71 [0·84-8·76] |  |  |
|  |  |  |  |  |  |  |  |
| Paris | 2·47 [1·60-3·81] |  | 1·03 [0·33-3·18] |  | 3·12 [1·98-4·92] |  |  |
| Val de Marne | 1·24 [0·82-1·89] |  | 0·19 [0·02-1·70] |  | 1·51 [0·97-2·34] |  |  |
|  |  |  |  |  |  |  |  |
| Bologna | 1·62 [1·05-2·51] |  | 1·03 [0·50-2·14] |  | 2·00 [1·25-3·20] |  |  |
| Palermo | 2·29 [1·34-3·91] |  | 1·66 [0·55-5·02] |  | 2·53 [1·43-4·46] |  |  |
|  |  |  |  |  |  |  |  |
|  |  |  |  |  |  |  |  |
| Interaction of | 23·9/ 12/ 0·0208^3)^ |  | 15·0/ 12/ 0·2439 |  | 20·1/ 12/ 0·0651 |  |  |
| {site x migration} |  |  |  |  |  |  |  |
|  |  |  |  |  |  |  |  |
| Pooled IRR_adj_ ^4)^ | 1·88 [1·65-2·14] |  | 1·14 [0·91-1·42] |  | 2·32 [2·03-2·66] |  |  |
|  |  |  |  |  |  |  |  |

^1)^ Including Europe, USA, Canada, USA, Australia, New Zealand and countries of former Soviet Union, except states in Asia with a predominantly Islamic population; ^2)^ Middle East, The Maghreb, sub-Saharan Africa, Asia, The Caribbean (including French oversees departments), Latin America; ^3)^ Wald χ2 statistic/ df/ P value; ^4)^ adjusted for age, gender, and recruitment site

**eTable 5. Age- and gender-adjusted Incidence rate ratios (IRR_adj_) of NAPD, for certain non-Western migrant and minority ethnic groups (“minorities”), compared to the local reference population, by site of recruitment.**

|  | **Minorities from:** |  |  |  |  |  |
| --- | --- | --- | --- | --- | --- | --- |
|  | **Middle East^1)^** | **The Magreb^2)^** | **sub-Saharan Africa** | **Asia^3)^** | **The Caribbean^4)^** | **Latin America** |
|  | **IRR_adj_ [95%-CI]** | **IRR_adj_ [95%-CI]** | **IRR_adj_ [95%-CI]** | **IRR_adj_ [95%-CI]** | **IRR_adj_ [95%-CI]** | **IRR_adj_ [95%-CI]** |
|  |  |  |  |  |  |  |
| London | 9·09 [4·84-17·09] |  | 3·54 [2·36-5·32] | 1·28 [0·72-2·25] | 3·41 [2·19-5·31] |  |
| Cambridge | 2·09 [0·33-13·34] |  | 5·97 [2·72-13·11] | 1·82 [1·05-3·15] | 3·64 [1·23-10·84] |  |
|  |  |  |  |  |  |  |
| Amsterdam | 2·12 [1·16-3·89] | 4·23 [2·54-7·07] | 3·95 [2·29-6·83] | 1·69 [0·90-3·19] | 3·10 [2·08-4·60] | 4·12 [2·00-8·49] |
| Gouda & Voorhout | 1·40 [0·37-5·22] | 3·81 [2·08-6·99] | 3·36 [0·89-12·60] | 1·07 [0·30-3·86] | 0·79 [0·09-6·73] | 7·01 [1·93-25·52] |
|  |  |  |  |  |  |  |
| Barcelona |  | 2·86 [1·06-7·72] | 1·92 [0·38-9·60] | 0·42 [0·12-1·52] | 0·00 [-] | 1·12 [0·51-2·46] |
| Valencia |  | ↑^6)^ | ↑ | ↑ | ↑ | 1·24 [0·44-3·51] |
| Oviedo |  | 5·40 [1·78-16·36] | 0·00 [-] | 3·02 [0·33 – 27·22] | ↑ | 3·07 [1·18-8·03] |
| Santiago |  | ↑ | ↑ | ↑ | ↑ | 0·00 [-] |
| Cuenca |  | ↑ | ↑ | ↑ | ↑ | 2·50 [0·49-12·77] |
|  |  |  |  |  |  |  |
| Paris |  | 2·05 [1·03-4·11] | 4·52 [2·56-8·01] |  | 3·23 [1·07-9·76] |  |
| Val de Marne |  | 1·01 [0·47-2·16] | 2·50 [1·43-4·37] |  | 0·85 [0·28-2·52] |  |
|  |  |  |  |  |  |  |
| Bologna | 0·00 [-] | 3·70 [1·66-8·27] | 2·49 [0·79-7·90] | 1·76 [0·95-3·25] |  | 0·80 [0·09-7·31] |
| Palermo | ↑ | 1·14 [0·13-10·08] | 5·48 [2·41-12·47] | 1·70 [0·68-4·24] |  | 2·20 [0·24-19·98] |
|  |  |  |  |  |  |  |
|  |  |  |  |  |  |  |
| Interaction of | 13·5/ 4/ 0·0091^5)^ | 13·4/ 7/ 0·0636 | 5·9/ 9/ 0·7503 | 5·6/ 7/ 0·5821 | 7·1/ 6/ 0·3107 | 11·1/ 8/ 0·1939 |
| {site x migration} |  |  |  |  |  |  |
|  |  |  |  |  |  |  |
| Pooled IRR_adj_ ^7)^ | 2·75 [1·79-4·23] | 2·68 [2·03-3·54] | 3·56 [2·82-4·49] | 1·43 [1·08-1·87] | 2·65 [2·07-3·4] | 1·94 [1·26-2·98] |
|  |  |  |  |  |  |  |

^1)^  Turkey, Israel, Egypt, Iran, Iraq and other countries in the region; UK: self-identified Arabs; ^2)^North-African countries, except Egypt; ^3)^including those states of the former Soviet Union with a predominant Islamic population; ^4)^Surinam, Guyana, and French Guyana and the other French overseas departments; ^5)^ Wald χ2 statistic/ df/ P value; ^6)^↑ site collapsed with the preceding one; ^7)^ adjusted for age, gender, and recruitment site

References

Andreasen, N. C., Flaum, M., & Arndt, S. (1992). The Comprehensive Assessment of Symptoms and History (CASH). An instrument for assessing diagnosis and psychopathology. *Archives of General Psychiatry, 49*(8), 615-623.

Nurnberger, J. I., Jr., Blehar, M. C., Kaufmann, C. A., York-Cooler, C., Simpson, S. G., Harkavy-Friedman, J., . . . Reich, T. (1994). Diagnostic interview for genetic studies. Rationale, unique features, and training. NIMH Genetics Initiative. *Archives of General Psychiatry, 51*(11), 849-859; discussion 863-844.

World Health Organization (1992). Schedules for Clinical Assessment in Neuropsychiatry. Geneva, Switzerland: World Health Organization.
